# A Reproducible, Data‐Driven Approach to Mapping Species Distributions Using Presence‐Only Data and Biogeographic Templates

**DOI:** 10.1002/ece3.72285

**Published:** 2025-10-10

**Authors:** Cristian S. Montalvo‐Mancheno, Jessie C. Buettel, Stefania Ondei, Barry W. Brook

**Affiliations:** ^1^ School of Natural Sciences University of Tasmania Sandy Bay Australia; ^2^ ARC Centre of Excellence for Australian Biodiversity and Heritage (CABAH) University of Tasmania, School of Natural Sciences, University of Tasmania Hobart Australia

**Keywords:** biogeographic regionalization, conservation biogeography, kernel density estimation, macroecology, presence‐only data, species distribution

## Abstract

Expert‐derived range maps are used extensively in macroecological and biogeographic analyses, yet they are subjective, taxonomically biased, and inconsistent in their treatment of species' absences. We developed a reproducible, data‐driven approach to estimate species' extent of occurrence (EOO) using presence‐only data and subregions of the Interim Biogeographic Regionalization for Australia (IBRA). This approach employs a Gaussian kernel density estimator calibrated for spatial coherence and ecological realism, producing maps independent of arbitrary grid structures. We applied it to 610 Australian bird species and evaluated the concordance of our data‐driven EOO maps against BirdLife International expert‐derived range maps. The spatial association between these two map sources, measured on a 0–1 scale, ranged from near‐zero to 0.93 across species, with higher concordance among terrestrial birds. Estimates of richness using both map sources varied most at the finest spatial scale (IBRA subregions), with mean and root mean square errors at the coarsest biogeographic scale (ecoregion) being 1/3 lower than those at the subregional scale. Likewise, we demonstrated the approach's applicability across taxa by generating data‐driven EOO maps for selected amphibians, mammals, reptiles, and vascular plants. Like for birds, discontinuities in the distribution of these non‐avian species represented different subpopulations over Australia. Our approach minimizes the influence of sampling bias and internal discontinuities in the estimation of species' EOO, while allowing for range edge refinement and subpopulation delineation. It provides an objective and scalable alternative to expert‐derived range maps, well‐suited for large‐scale ecological research requiring consistency in spatial precision. Given the plethora of biogeographic templates already in use, our approach is adaptable to many contexts and thus can readily support a better understanding and conservation of biodiversity at large spatial scales.

## Graphical abstract

This study presents a novel, data‐driven approach for mapping species' extent of occurrence (EOO) that addresses the issues of reproducibility and precision of expert‐derived range maps. By combining presence‐only data with the operational units of a biogeographic template within a consistent quantitative framework, our approach generates spatially coherent range maps for large‐scale biodiversity research. Demonstrated using Australia's native terrestrial species, this approach offers a scalable, practical tool for biodiversity research and conservation at macroscales, while also providing an objective basis for deriving finer‐scale metrics of species' range size across diverse taxonomic groups and geographic regions.

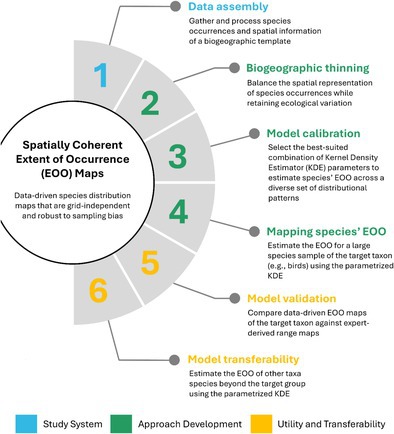

## 1 Introduction

Species occurrence data and range maps are important pillars in our understanding of the geography of biodiversity, making both occupancy data sources essential for biogeographic and macroecological studies (Jetz et al. 2012). Occurrences have been used, for example, to assess the impact of global environmental change on species, to identify sites of conservation priority, and to map species ranges. Although occurrences have also been used to reveal biogeographic patterns (e.g., Bloomfield et al. 2018; Ondei et al. 2019; Smith et al. 2018), range maps are increasingly recognized as quantitative spatial objects appropriate for large‐scale studies (Marsh et al. 2022). Consequently, range maps have been used to investigate taxonomic richness and composition, niche attributes, and range‐size dynamics (Jetz and Rahbek 2002; Kreft and Jetz 2010; Lyons et al. 2019; Olalla‐Tárraga et al. 2011). They have further been linked with other biodiversity facets (e.g., phylogenetic) to examine the forces shaping zoogeographic regions (Ficetola et al. 2017; Holt et al. 2013) and to evaluate the variation in functional and phylogenetic diversity (Jarzyna et al. 2021; Maestri and Duarte 2020). Range maps and occurrence data have further application in systematic conservation planning (Lamoreux et al. 2006; Pacifici et al. 2018) and extinction risk assessment (Bachman et al. 2019; Brooks et al. 2019). While both occupancy data sources are increasingly available over larger regions and finer resolutions, they still suffer from taxonomic bias and methodological limitations (Jetz et al. 2012).

One persistent issue is that species occurrences are heavily biased toward areas more easily accessible (e.g., roads, visitor centers, tracks, etc.), with a greater number of observations of diurnal, conspicuous, charismatic, and easy to identify species. Minimizing the pervasive effect of sampling bias is not a trivial task, and multiple methods have been developed to address this issue (Aiello‐Lammens et al. 2015). One option is to manipulate background data to mirror the bias structure, for example, by constructing bias grids, restricting backgrounds to target groups, or weighting samples by effort (Barber et al. 2022; Dudík et al. 2007; Phillips et al. 2009). Another is to manipulate occurrences directly, with thinning being the most applied strategy. Spatial thinning filters occurrences in the geographic space based on either a stratified sample (e.g., one occurrence per grid cell) or a minimum nearest neighbor distance constraint (Boria et al. 2014; Pearson et al. 2007), while environmental thinning filters occurrences based on environmental characteristics (Castellanos et al. 2019; Varela et al. 2014). However, recent work has shown that removing nearby occurrences often fails to improve model accuracy (Ten Caten and Dallas 2023) and that no universal optimal filtering distance exists (Lamboley and Fourcade 2024). Because bias correction is influenced by numerous correlates of sampling effort (e.g., the extent and resolution of analysis, and the number and spatial arrangement of occurrences), the empirical determination of the optimal degree of thinning is largely considered a goal‐oriented rather than theory‐driven task, where each method trades off reproducibility, data loss, and uncertainty.

Range maps represent the areas where a species is known or expected to occur during its lifespan. These maps have been derived using deductive, transparent, and reproducible methods (Gaston and Fuller 2009; Jetz et al. 2012; Marsh et al. 2022). Two of the most widely used range metrics are the extent of occurrence (EOO) and the area of occupancy (AOO). Although both quantify fundamentally different aspects of a species' range (Gaston and Fuller 2009), AOO is often perceived as more ecologically representative than EOO due to its ability to account for internal patchiness and unoccupied areas within the overall geographic extent (Hurlbert and Jetz 2007; Jetz et al. 2008; Shaw et al. 2003). Notably, most currently available range maps are, to some degree, the product of the tacit knowledge of experts who have compiled occurrences alongside ancillary data to define the regions a species lives (Marsh et al. 2022). Due to the array and disparity of data sources and types, expert‐derived range maps differ in quality both among species and between regions for a given species (Marsh et al. 2022; Palacio et al. 2021). These maps are prone to commission and omission errors (Gaston 1991; Graham and Hijmans 2006; Mainali et al. 2020; Peterson et al. 2018), with their accuracy being especially compromised at finer spatial resolutions and influenced by ecological attributes (Ficetola et al. 2014; Hurlbert and Jetz 2007; Hurlbert and White 2005; Jetz et al. 2008). Much of this inconsistency also arises from mapping precision, defined as how experts balance the level of spatial detail given to the edges of a species' range and the identification of its different populations (Marsh et al. 2022).

The era of big data in ecology has facilitated the estimation of species distributions at higher resolution and with greater accuracy (Brooks et al. 2019; García‐Roselló et al. 2019; Palacio et al. 2021). Species distribution modeling is a valuable tool for estimating the EOO and AOO of a species over large areas (IUCN Standards and Petitions Committee (IUCN) 2022). Applying a threshold to the predicted continuous values on a gridded output of these models is an unavoidable step. The reliability of those range maps thus depends on modeling decisions that are species‐ and system‐specific, making consistent application across multiple species a significant challenge (Palacio et al. 2021). When carefully tuned, modeled range maps have proven useful for applied purposes such as conservation planning and decision‐making at a local scale (Breiner et al. 2017; Pena et al. 2014; Syfert et al. 2014). But for large‐scale biodiversity studies, coarser spatial resolutions are advised (Hurlbert and Jetz 2007). Additionally, aggregating ecological phenomena using uniform grid cells can introduce spatial artifacts stemming from the size, shape, and positioning of the grid units (Heywood et al. 2011; Moat et al. 2018). Empirical and simulated studies have shown that even small variations in the grid's origin can lead to substantial uncertainty in species range estimates (Breiner and Bergamini 2018; Keith et al. 2018; Moat et al. 2018). One alternative is to bypass arbitrary grid structures by applying interpolation techniques to presence‐only data within a spatial framework defined by environmentally coherent, non‐equal‐sized operational units (e.g., biogeographic templates), with kernel‐based methods in particular being widely used to map diversity and areas of endemism and often outperforming grid‐based methods by capturing fuzzy range edges and overlap among species (Biondi et al. 2021; Oliveira et al. 2015).

**FIGURE 1:**
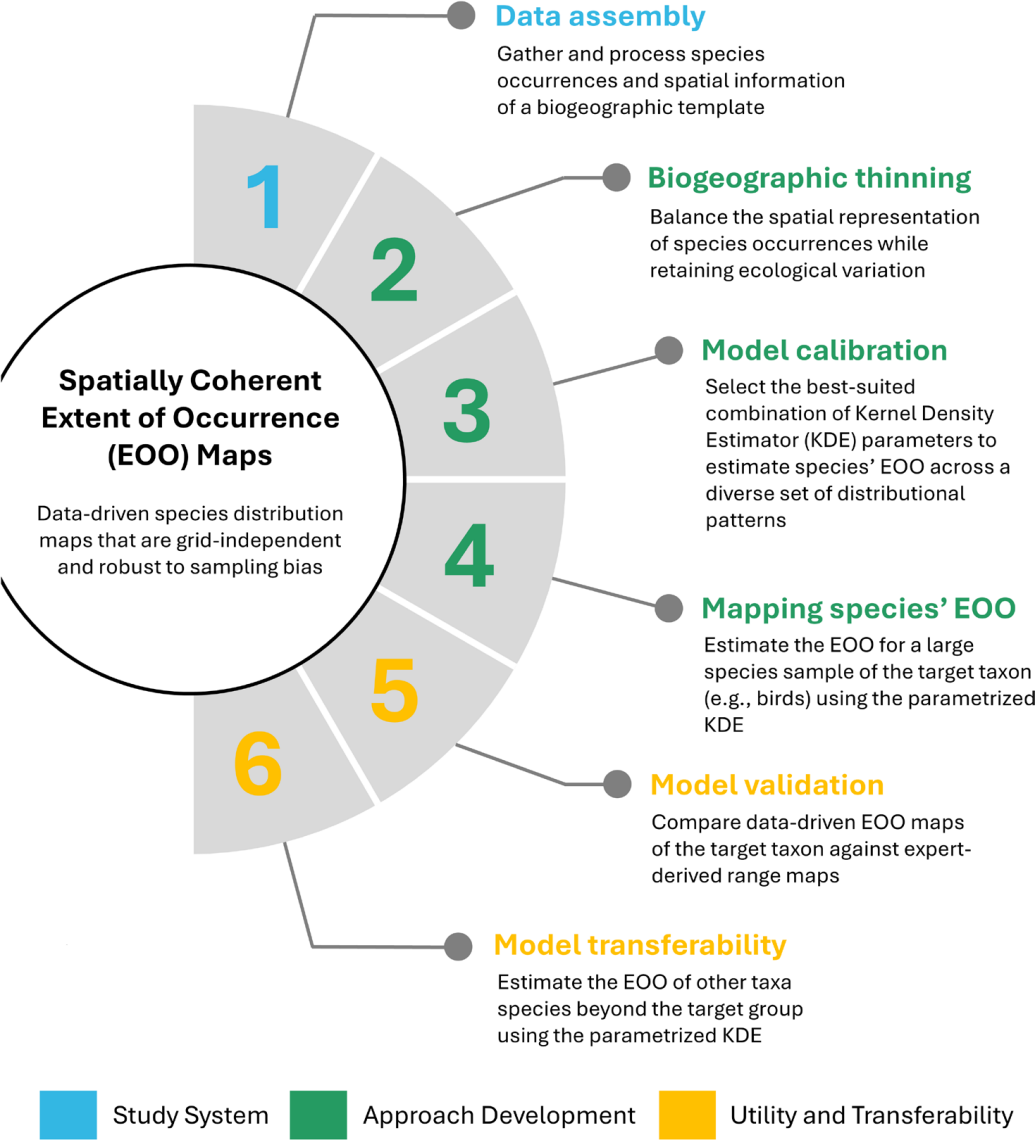
Deriving spatially coherent extent of occurrence maps. Details of the approach are described in the following sections: Study System, Approach Development, and Utility and Transferability.

Our goal is to provide a reproducible, scalable, and objective alternative to expert‐derived maps. The approach maps species' EOO by combining presence‐only data with the operational units of a biogeographic template within a unifying quantitative framework to minimize inconsistencies in spatial precision (Figure 1). Using Australia as a model, we:

1. Gather and process species occurrences and spatial information of a biogeographic template (Study System);
2. Define data requirements and kernel‐density parameters for objectively estimating species' EOO for wide‐ranging, habitat‐specialist, and range‐restricted birds (Approach Development); and
3. Evaluate the value and generality of the approach by comparing data‐driven EOO maps with expert‐derived range maps across a large bird sample, and by testing its transferability to amphibians, mammals, reptiles, and vascular plants (Utility and Transferability).

## 2 Study System

### 2.1 Case Study Context

Australia is an ideal study system for developing and evaluating the proposed approach due to its well‐documented biota and wide climatic and ecological variation, spanning from deserts to tropical forests. It is among the world's ten most biodiverse countries (Mittermeier et al. 1997) but the sole country with a comprehensive inventory of species occurrences across taxa, databased within a centralized, open‐access repository (e.g., the Atlas of Living Australia). While being well‐studied taxa in other regions, birds and mammals are also both conspicuous and vulnerable to extinction in Australia (Johnson 2006). Moreover, as an island continent, Australia has natural oceanic boundaries and a clearly defined biogeographic framework: the Interim Biogeographic Regionalization for Australia (IBRA; Thackway and Cresswell 1995).

### 2.2 Datasets

We downloaded species occurrences from the spatial portal of the Atlas of Living Australia (ALA; Belbin 2011), including all birds native to Australia and two species each of amphibians, mammals, reptiles, and vascular plants (Details of data usage are provided in Sections 3 and 4). Across taxa, we applied the preprocess protocol of Ondei et al. (2019), excluding occurrences without geographic coordinates, dated before 1990, or outside the geographic extent of the study system. For non‐avian taxa, we selected species with well‐documented distributions and occurrences spanning the core of their Australian range, covering mainland Australia and/or the main island of Tasmania, to ensure that the resulting EOO maps were ecologically sensible.

Defined by the Australian Department of Agriculture Water and the Environment (ESRI) (2012a), the IBRA framework is the first and most detailed biotic regionalization of Australia (Ebach 2012). It is one of the few systems directly aligned with WWF's terrestrial ecoregions (Olson et al. 2001), a widely applied global biogeographic template. The framework consists of a nested hierarchy of biogeographic units, with smaller areas demarcating major regional ecosystems (subregions) and the larger ones clustering repeating assemblages of interacting ecosystems (bioregions). We sourced the IBRA framework from version 7 of the subregion's dataset in shapefile format (Department of Agriculture Water and the Environment (ESRI) 2012b), which includes 419 operational units. After excluding oceanic islands and islets (< 1 km^2^ landmass), we retained 410 IBRA subregions nested within 85 IBRA bioregions, themselves nested within 37 WWF ecoregions. This three‐tier hierarchy reflects IBRA's role as a detailed geographic classification of the WWF's ecoregions, enabling our comparison between data‐driven EOO maps and expert‐derived range maps to be based on the most up‐to‐date, regional‐to‐global framework for Australia's biodiversity conservation.

We carried out all spatial processing in ArcMap (Environmental Systems Research Institute (ESRI) 2017) using the Australian Albers Equal Area projection (EPSG 3577). We completed all spatial analysis and feature engineering in R (R Core Team 2020), using alongside the specific packages noted in the subsequent sections the following libraries: sf (Pebesma 2018), spatialEco (Evans 2019), parallel (R Core Team 2020), tidyverse (Wickham et al. 2019), reshape2 (Wickham 2007), lwgeom (Pebesma 2019), and magrittr (Bache and Wickham 2014).

## 3 Approach Development

The kernel‐density estimator (KDE) is a well‐established technique for estimating species' EOO. It is widely applied to space‐related problems across ecology, evolution, and biogeography, supported by a rich statistical literature (Fleming and Calabrese 2017). However, reliable results from KDE require two key components: (i) sufficient, spatially unbiased data, and (ii) appropriate control over the precision of boundary estimation. We addressed both by structuring the development of this approach in two parts. First, we homogenized sampling effort across the biogeographical space and defined a minimum‐sample‐size threshold. We then calibrated how roughly the range boundary is approximated and how precisely the spread of species' localities is delineated.

### 3.1 Data Requirements

To even out sampling efforts across landscapes, we used IBRA subregions as our standard units of analysis. We started by classifying subregions based on log‐transformed area into three size classes: small, medium, and large. For this classification, we used the Jenks natural‐breaks optimization algorithm (Jenks 1977; Jenks and Caspall 1971), implemented via the BAMMtools R package (Rabosky et al. 2014). We capped the number of species occurrences at the mean for each size category and sampled them without replacement. This procedure, hereafter referred to as “biogeographic thinning”, aims to balance spatial representation while retaining ecological variation. We then set a minimum threshold of 20 occurrences per species after biogeographic thinning, which aligns with the theoretical minimum sample size needed to maintain a relative mean square error less than 0.1 when estimating a bivariate density with a kernel function (Silverman 1986).

### 3.2 Calibrating the Kernel Density Estimator (KDE)

We assessed the 95% and 99% confidence regions of utilization distributions for two KDE smoothing parameters: reference bandwidth and plug‐in estimator, implemented in the adehabitatHR (Calenge 2006) and ks (Duong 2019) R packages, respectively. We aimed to examine the sensitivity of KDE outputs to parameter selection and determine whether the method could reliably estimate EOO for three broad types of distributions: wide‐ranging, habitat‐specialist, and range‐restricted species. We chose birds as the test group because they are conspicuous, widely studied in macroecology, and benefit from rich open‐source datasets across many regions. We used an example set of six bird species, with each species pair representing one of the above distribution types, and applied biogeographic thinning as described in Section 3.1 before deriving the EOO maps.

To select the best‐suited KDE parameters combination (i.e., least prone to overestimate species' utilization distributions), we visually inspected the spatial coherence of resulting EOO maps within and across the example set of birds. Based on these visual checks and following the recommendation to avoid mapping disjunctions when measuring EOO (Gaston and Fuller 2009), we chose the plug‐in bandwidth with a 99% confidence region as the best‐suited parameter combination (see Section 5.1 for results). This configuration was then used for all subsequent EOO mapping.

## 4 Utility and Transferability

We tested the value of our approach in two ways: (i) by comparing data‐driven EOO maps to expert‐derived range maps for a large sample of birds, focusing on the degree of spatial association between these maps and the difference in species richness at multiple biogeographic scales, and (ii) by exploring whether the approach could be applied to other taxa besides birds.

### 4.1 Comparison Between Data‐Driven and Expert‐Derived Maps of Birds

Of the 1292 native birds with georeferenced occurrences in the ALA's dataset, 815 met the minimum sample size threshold (i.e., ≥ 20 occurrences after biogeographic thinning; Section 3.1). Using the optimal KDE parameter configuration (i.e., plug‐in bandwidth and 99% confidence region; Section 3.2), we derived data‐driven EOO maps for 672 species and 143 subspecies. We sourced expert‐derived range maps from BirdLife International (BirdLife International and Handbook of the Birds of the World 2021), retaining only those overlapping with the Australian study extent. Each of the 832 expert‐derived maps consists of one or more polygons with information about a species' distributional attributes, which were used to exclude polygons marked as ‘locally extinct’. Since the ALA dataset includes species and subspecies, whereas BirdLife only covers species, the difference in the number of species counts is expected. Then, to ensure comparability, we matched scientific names between both datasets using the Integrated Taxonomic Information System via the taxize R package (Chamberlain and Szöcs 2013). This yielded a final sample of 610 bird species represented in both datasets (Table S1).

We assessed the degree of spatial association between map types by computing the ‘V‐measure’, a global metric grounded in information theory and interpretable in terms of analysis of variance (Nowosad and Stepinski 2018). V‐measure values range from 0 (totally different distributional pattern) to 1 (identical distributional pattern), allowing us to quantify the amount of shared information between pairs of maps for each species. We first generated presence‐absence maps for all birds from each dataset and then used the saber R package (Nowosad and Stepinski 2018) to compute the V‐measure for each species. We summarized the results by plotting the distribution and reporting the mean and standard deviation across all birds (*n* = 610), and again after excluding shore and pelagic birds (*n* = 495), which excluded species from the Charadriiformes, Procellariiformes, and Sphenisciformes orders.

To examine how differences in both sources of distributional data (i.e., data‐driven EOO and expert‐derived maps) can affect species richness, we derived presence‐absence matrices at three biogeographic scales: IBRA subregions (*n* = 410), IBRA bioregions (*n* = 85), and WWF ecoregions (*n* = 37). For each biogeographic unit, we calculated the total number of birds using both map sources. We then computed the root mean square error (RMSE) of the difference between species richness from the data‐driven EOO maps and that from the expert‐derived range maps as a measure of variation, and the mean error for bias. Finally, we plotted variation and bias in species richness to visually summarize the results of this analysis.

### 4.2 Transferability to Other Taxa

To test the generality of the approach, we applied it to one species pair from each of the following taxa: amphibians, mammals, reptiles, and vascular plants, all native to Australia. Using the same KDE configuration as for birds, we visually examined the spatial coherence of each species' data‐driven EOO map by itself and within the broader taxonomic groups, by looking for signs that the edges of its utilization distribution and/or the spread of its population localities were poorly represented at the bioregional scale. We conducted all analyses and statistical summaries in R (R Core Team 2020), with maps prepared in ArcMap (Environmental Systems Research Institute (ESRI) 2017).

## 5 Results

### 5.1 Determining the Best‐Suited Parameter Combination

**FIGURE 2:**
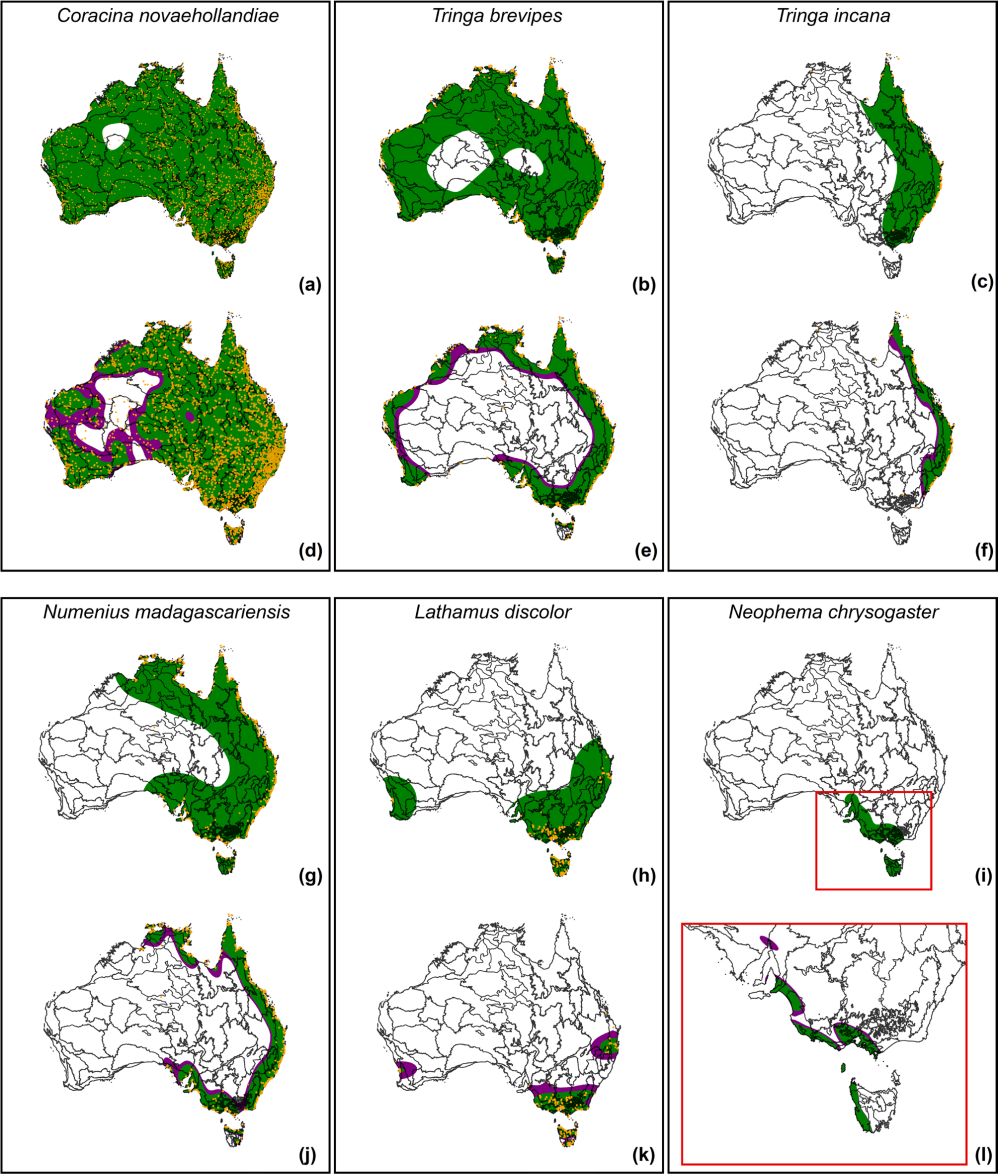
Extent of occurrence maps for six bird species used to calibrate the Kernel Density Estimator (KDE) parameters. Each map shows the overlap between Australia's bioregions and species' utilization distributions estimated with a Gaussian KDE using either the reference bandwidth (a–c, and g–i) or the plug‐in estimator (d–f, and j–l). Green and purple polygons represent the 95% and 99% confidence regions, respectively.

Our preliminary examination showed that the reference bandwidth consistently overestimated species' EOO, even when using a 95% confidence region. For the example set of six birds, the resulting EOO maps extended well beyond realistic range boundaries (Figure 2a–c,g–i), signaling that this smoothing parameter poorly constrained the edges of the utilization distribution. Yet, this was not the case when using the plug‐in bandwidth estimator, meaning that, based on visual checks, this smoothing parameter produced more spatially coherent EOO maps for our example set of birds.

We compared two confidence regions (95% or 99%) applied to KDEs using the plug‐in estimator. The 95% region tended to produce disjunctions in the estimated utilization distributions (Figure 2d–f,j–l). These ‘holes’ in EOO maps may obscure broader distributional patterns and misrepresent species' presence. Conversely, the 99% confidence region reduced disjunctions in the estimated utilization distributions while still excluding potential outliers, which might represent vagrant or misidentified species. While in some cases, the EOO maps using both confidence regions differed little (Figure 2f,l), the 99% region generally produced more spatially cohesive results. Therefore, we adopted the plug‐in bandwidth with a 99% confidence region as the most suitable parameter combination for all subsequent EOO mapping.

### 5.2 Spatial Agreement Between Data‐Driven EOO Maps Expert‐Derived Maps

**FIGURE 3:**
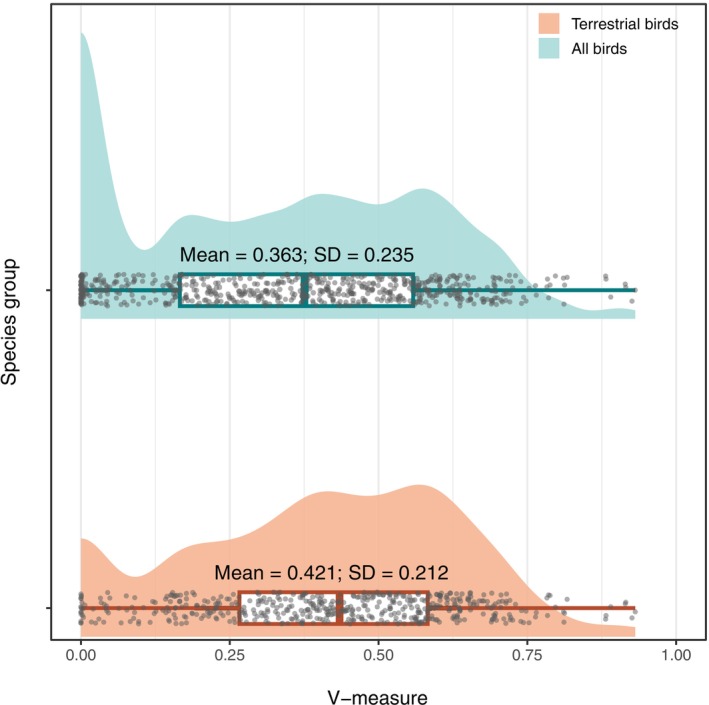
Distribution of the degree of spatial association between data‐driven EOO and expert‐derived range maps. Each gray dot represents the V‐measure comparing presence‐absence maps for an individual bird species using both map sources. The upper panel shows results for all bird species (*n* = 610), while the lower panel shows results for terrestrial birds (*n* = 495), which excludes species from the Charadriiformes, Procellariiformes, and Sphenisciformes orders. The V‐measure ranges from 0 (i.e., totally different distributional pattern) to 1 (i.e., identical distributional pattern). For species groups, mean and standard deviation (SD) are reported.

The spatial association between data‐driven EOO maps and expert‐derived range maps varied widely across bird species (Figure 3). Using the V‐measure to quantify the amount of shared information regarding species' presence and absence between map sources, we found values ranging from near zero (2.3 × 10^−7^; indicating minimal spatial concordance) to nearly one (0.93; indicating an almost identical spatial pattern). For all 610 bird species, the distribution of V‐measure scores was broad. When we focused on terrestrial birds (*n* = 495), which excluded species from the Charadriiformes, Procellariiformes, and Sphenisciformes orders, the spread of V‐measures narrowed, and the mean spatial association was higher (Figure 3).

### 5.3 Estimates of Species Richness at Multiple Biogeographic Scales

**FIGURE 4:**
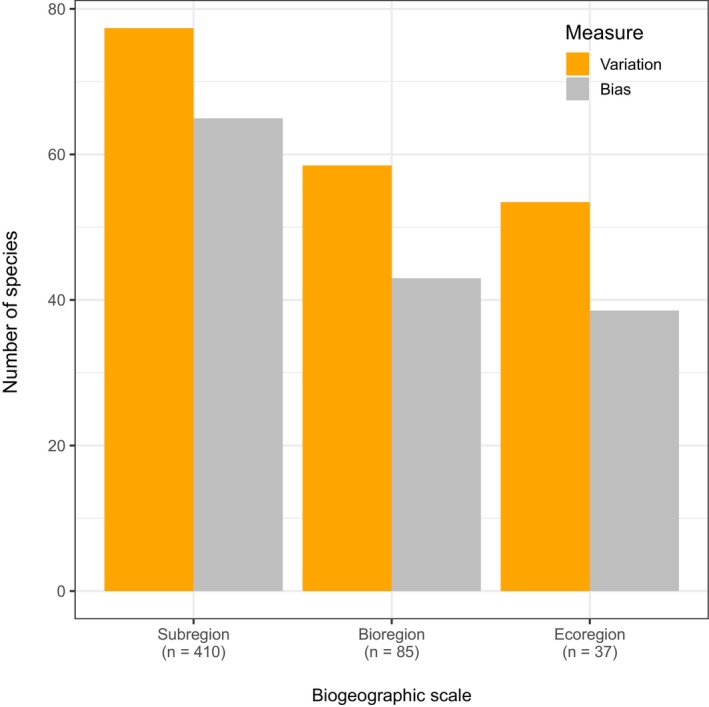
Comparison of species richness estimates across biogeographic scales. Variation is represented by the root mean square error and bias by the mean difference in species richness estimates between the data‐driven EOO maps and expert‐derived range maps. The *y*‐axis for bias could in theory range from −610 to 610 (i.e., all bird species).

Differences in species richness estimates between map sources were evident at the magnitude of both variation and bias across all three biogeographical scales, with higher richness values resulting from data‐driven EOO maps (Figure 4). At the finest scale, IBRA subregions, species richness estimates varied most, as indicated by RMSE. This variation decreased progressively at broader scales, with the lowest RMSE observed at the ecoregional scale. The same trend was seen in bias, measured as the mean error in species richness estimates. As expected, the discrepancy in estimates decreased at coarser resolutions, with bias at the ecoregional scale being about 1/3 lower than that at the subregional scale (Figure 4).

### 5.4 Transferability to Other Taxa

**FIGURE 5:**
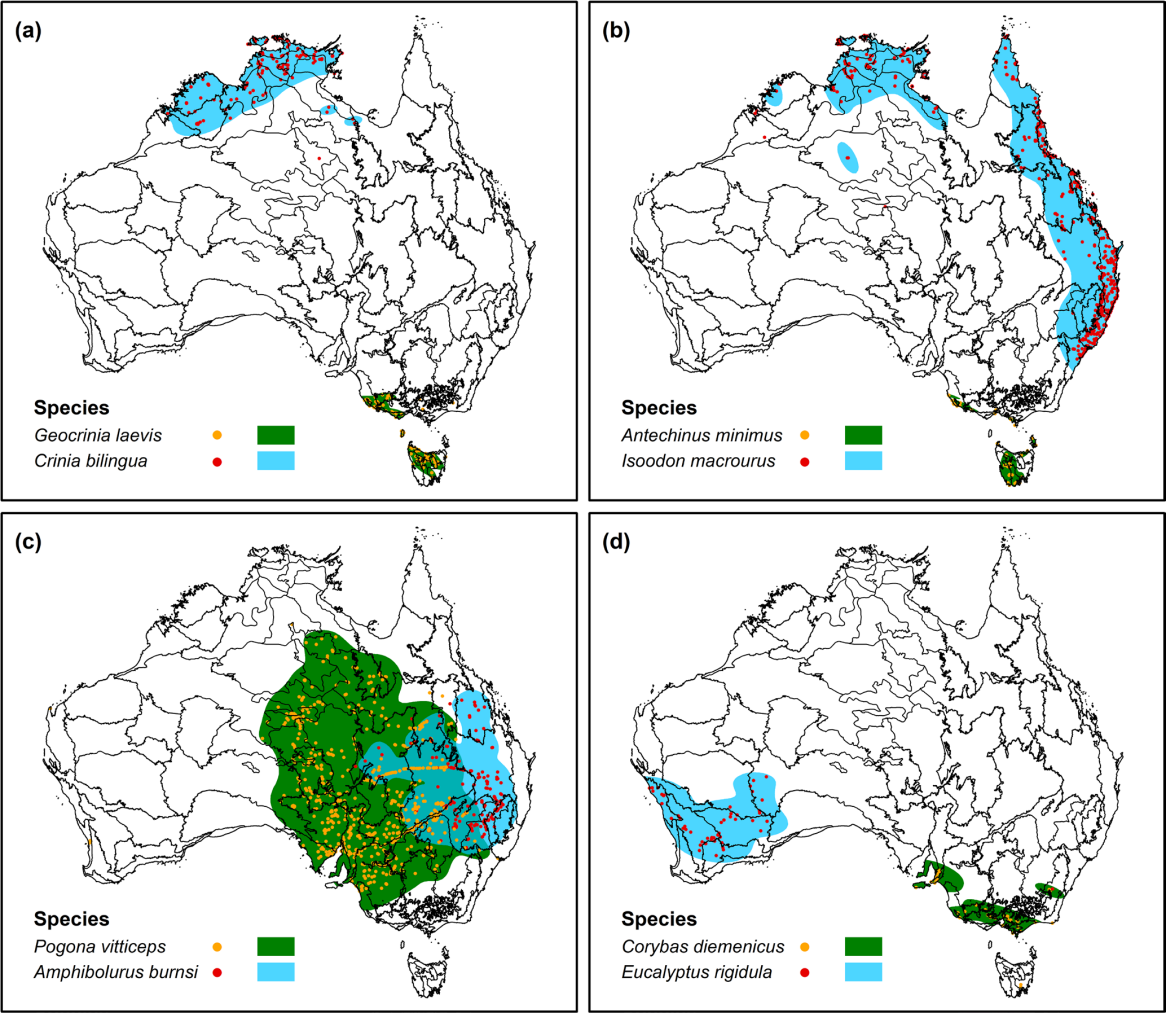
Data‐driven extent of occurrence (EOO) maps for selected non‐avian taxa. EOO maps are shown for one representative species each from (a) amphibian, (b) mammal, (c) reptile, and (d) vascular plants (species). Each map shows the overlap between the species' utilization distributions and Australia's bioregions. EOO estimates were generated using a Gaussian kernel density function with the plug‐in estimator and 99% confidence region.

Like with the example set of six birds (Figure 2), two patterns were also consistent across the data‐driven EOO maps for one pair of amphibians, mammal, reptile, and vascular plant species (Figure 5). First, disjunctions or ‘holes’ in EOO maps of these non‐avian species appeared to reflect the demarcation of different populations across Australia rather than the underestimation of the species' utilization distributions. Second, it seems that the estimation of these species' EOO was able to systematically deal with outlier occurrences, which likely correspond to vagrant individuals or misidentifications (Figure 2).

## 6 Discussion

Despite their subjectivity, expert‐derived range maps are increasingly treated as quantitative spatial data suitable for macroecological and biogeographic analyses (Marsh et al. 2022). Conversely, data‐driven range maps have proven useful for applied and local‐scale purposes due to their higher resolution and greater accuracy (Palacio et al. 2021). Nonetheless, their reliance on a uniform grid structure can introduce artifacts linked to the size, shape, and placement of cells (Heywood et al. 2011; Moat et al. 2018). In this study, we addressed these limitations by developing a reproducible, data‐driven approach to mapping the extent of occurrence (EOO) of hundreds to thousands of species at coarse resolutions. Our approach integrates presence‐only data and subregions (i.e., the finest resolution of the IBRA framework) within a unifying quantitative framework. Our findings demonstrate that this approach provides an objective, scalable, data‐driven alternative to expert‐derived range maps (Figures 3, 4, 5). It can reliably generate coherent EOO maps that are independent of arbitrary grids across three broad distributional patterns: wide‐ranging, habitat‐specialist, and range‐restricted species.

There is no universal approach for estimating species' range sizes that suits all ecological and analytical purposes (Jetz et al. 2008; Qiao et al. 2015). Estimates of species diversity within spatial units of analysis (e.g., bioregions) can vary depending on the range maps used as sources of information, with such discrepancies decreasing as the spatial unit size increases (Hurlbert and Jetz 2007). For example, differences in richness estimates between two sources of expert‐derived mammal range maps reached up to 158 species at a 0.5‐degree grid resolution (Marsh et al. 2022). Our comparison of data‐driven EOO and expert‐derived maps for Australian birds shows a similar pattern (Figure 4). These mismatches in richness estimates are likely driven by how species' absences are inferred, particularly the approximation of range edges and delineation of subpopulations (i.e., precision; Marsh et al. 2022). This is evident in the marked differences in spatial association between presence‐absence patterns derived from data‐driven EOO and expert‐derived range maps (Figure 3). Additional discrepancies may stem from differences in coastline mapping, especially for shorebirds and pelagic species, and from the exclusion of large freshwater bodies in expert‐derived but not in data‐driven EOO maps. Consequently, it is impractical to claim that one set of maps is superior to another (*sensu* Marsh et al. 2022), nor is that the aim of this study. Rather, we argue that the confounding effects introduced by inconsistent levels of precision in expert‐derived maps can be minimized by applying a standardized level of precision across species and taxa, ultimately improving our understanding of large‐scale biodiversity patterns.

Expert‐derived range maps are known to perform poorly for grid‐based analyses at resolutions finer than ~200 km due to the scale dependence in ecological processes (Hurlbert and Jetz 2007). They also suffer from inconsistent precision in boundary delineation and subpopulation identification (Marsh et al. 2022), potentially distorting large‐scale biodiversity estimates and, by extension, conservation priorities. The observed discrepancies in richness across biogeographical scales suggest that prior conclusions about richness‐environment relationships may have been inadvertently mischaracterized. If richness estimates are biased by such inconsistencies, then global and regional conservation priorities, such as key biodiversity areas and biodiversity hotspots, could have been misidentified or overemphasized, as they are often based on taxon richness derived from expert range maps. While not directly suitable for local applications, generating data‐driven EOO maps may serve as a more objective basis for deriving finer‐scale metrics of species range size (e.g., the area of occupancy and the habitat of occupancy).

We argue this approach offers a robust alternative for defining species' range boundaries in large‐scale biodiversity research and conservation planning. By anchoring EOO estimation to environmentally coherent, non‐equal‐sized operational units, it is adaptable to many contexts, drawing on the variety of biogeographic templates available across scales (Mackey et al. 2008). Additionally, data‐driven EOO maps vary little in quality and can readily account for recent changes in the geographic distribution of species, known shortcomings of expert‐derived range maps (Di Marco et al. 2017; Rondinini et al. 2011). Nonetheless, the utility of this approach depends on the quality of underlying occurrence data (Gaston and Fuller 2009; Marsh et al. 2022). Two caveats are particularly noteworthy. First, the accuracy of EOO estimation is influenced by biases in sampling effort and species' detectability. By standardizing sample effort across large regions and setting a minimum sample‐size threshold (Section 3.1), we mitigated potential mischaracterization of species' ranges at biogeographic scales. Second, our approach does not incorporate species absence data. While the inclusion of absences could improve range delimitation, such data are often scarce, even for well‐studied taxa like birds (Palacio et al. 2021), making absence‐inclusive EOO estimation impractical for large‐scale, multi‐species, multi‐taxa studies.

Future improvements could integrate data on biotic interactions and community structure, which are increasingly recognized as drivers of species distributions at large scale (Anderson 2017; Wisz et al. 2013). Likewise, advances in ecological datasets (Daru et al. 2017; Ficetola et al. 2017) and bioregionalization methods (e.g., Bloomfield et al. 2018; Coops et al. 2018; Edler et al. 2017; Holt et al. 2013; Maestri and Duarte 2020) offer promising pathways to refine this approach further. For instance, incorporating information on interspecific interactions, either explicitly in the estimation of species' EOO or implicitly through its use in the delineation of biogeographic operational units, could enhance the accuracy and ecological relevance of the results.

Using Australia and a representative set of its native species as a study system, this work demonstrates that spatially coherent, data‐driven EOO maps that are independent from an arbitrary grid structure can be reliably generated for many species across broad taxonomic groups. This is achieved through the combination of presence‐only data and non‐equal‐sized biogeographic units demarcating major regional ecosystems (e.g., 410 IBRA subregions) within a consistent quantitative framework. This approach addresses many shortcomings of expert‐derived range maps, offering a scalable alternative for robust, data‐driven analyses of biodiversity patterns. By improving the consistency and transparency of species' range mapping, this approach can support better‐informed conservation decisions and a clearer understanding of biodiversity patterns and processes at broad geographic scales.

## Author Contributions

**Cristian S. Montalvo‐Mancheno:** conceptualization (lead), data curation (lead), formal analysis (lead), investigation (lead), methodology (lead), visualization (lead), writing – original draft (lead), writing – review and editing (lead). **Jessie C. Buettel:** conceptualization (equal), investigation (equal), project administration (equal), supervision (supporting), writing – original draft (equal), writing – review and editing (equal). **Stefania Ondei:** conceptualization (equal), data curation (supporting), supervision (supporting), writing – original draft (equal), writing – review and editing (equal). **Barry W. Brook:** conceptualization (equal), formal analysis (equal), funding acquisition (lead), investigation (equal), methodology (equal), project administration (lead), supervision (lead), writing – original draft (equal), writing – review and editing (equal).

## Conflicts of Interest

The authors declare no conflicts of interest.

## Supporting information


**Table S1:** ece372285‐sup‐0001‐TableS1.docx.

## Data Availability

The datasets and code that support the findings of this study are available on Figshare at https://doi.org/10.6084/m9.figshare.24869112.
