# Supplementary material for: A Reproducible, Data‐Driven Approach to Mapping Species Distributions Using Presence‐Only Data and Biogeographic Templates
Source: Ecol Evol. 2025 Oct 10;15(10):e72285. doi: 10.1002/ece3.72285 (PMC12511788; doi:10.1002/ece3.72285)
Supplement: Supplementary file 1 — Table S1: ece372285‐sup‐0001‐TableS1.docx. [file ECE3-15-e72285-s001.docx]

**Table S1** Scientific names of the 610 bird species analyzed in the study, standardized following the Integrated Taxonomic Information System (ITIS).

| **Scientific name** |
| --- |
| *Acanthagenys rufogularis* |
| *Acanthiza apicalis* |
| *Acanthiza chrysorrhoa* |
| *Acanthiza inornata* |
| *Acanthiza iredalei* |
| *Acanthiza katherina* |
| *Acanthiza lineata* |
| *Acanthiza nana* |
| *Acanthiza pusilla* |
| *Acanthiza reguloides* |
| *Acanthiza robustirostris* |
| *Acanthiza uropygialis* |
| *Acanthorhynchus superciliosus* |
| *Acanthorhynchus tenuirostris* |
| *Accipiter cirrocephalus* |
| *Accipiter fasciatus* |
| *Accipiter novaehollandiae* |
| *Acrocephalus australis* |
| *Actitis hypoleucos* |
| *Aegotheles cristatus* |
| *Aerodramus terraereginae* |
| *Ailuroedus crassirostris* |
| *Alectura lathami* |
| *Alisterus scapularis* |
| *Amaurornis moluccana* |
| *Amytornis barbatus* |
| *Amytornis dorotheae* |
| *Amytornis goyderi* |
| *Amytornis merrotsyi* |
| *Amytornis modestus* |
| *Amytornis purnelli* |
| *Amytornis striatus* |
| *Amytornis textilis* |
| *Amytornis woodwardi* |
| *Anas castanea* |
| *Anas gracilis* |
| *Spatula rhynchotis* |
| *Anas superciliosa* |
| *Anhinga novaehollandiae* |
| *Anous minutus* |
| *Anous stolidus* |
| *Anseranas semipalmata* |
| *Anthochaera carunculata* |
| *Anthochaera chrysoptera* |
| *Anthochaera lunulata* |
| *Anthochaera paradoxa* |
| *Anthochaera phrygia* |
| *Anthus novaeseelandiae* |
| *Aphelocephala leucopsis* |
| *Aphelocephala nigricincta* |
| *Aphelocephala pectoralis* |
| *Aplonis metallica* |
| *Aprosmictus erythropterus* |
| *Apus pacificus* |
| *Aquila audax* |
| *Ardea alba* |
| *Bubulcus ibis* |
| *Ardea pacifica* |
| *Ardenna bulleri* |
| *Ardenna carneipes* |
| *Ardenna grisea* |
| *Ardenna pacifica* |
| *Ardenna tenuirostris* |
| *Ardeotis australis* |
| *Arenaria interpres* |
| *Arses kaupi* |
| *Arses lorealis* |
| *Arses telescopthalmus* |
| *Artamus cinereus* |
| *Artamus cyanopterus* |
| *Artamus minor* |
| *Artamus personatus* |
| *Artamus superciliosus* |
| *Ashbyia lovensis* |
| *Atrichornis rufescens* |
| *Aviceda subcristata* |
| *Aythya australis* |
| *Barnardius zonarius* |
| *Biziura lobata* |
| *Bolemoreus frenatus* |
| *Botaurus poiciloptilus* |
| *Burhinus grallarius* |
| *Butorides striata* |
| *Cacatua galerita* |
| *Cacatua pastinator* |
| *Cacatua sanguinea* |
| *Cacatua tenuirostris* |
| *Cacomantis castaneiventris* |
| *Cacomantis flabelliformis* |
| *Cacomantis variolosus* |
| *Calamanthus campestris* |
| *Calamanthus fuliginosus* |
| *Calidris acuminata* |
| *Calidris alba* |
| *Calidris canutus* |
| *Calidris ferruginea* |
| *Calidris melanotos* |
| *Calidris ruficollis* |
| *Calidris subminuta* |
| *Calidris tenuirostris* |
| *Caligavis chrysops* |
| *Callocephalon fimbriatum* |
| *Calonectris leucomelas* |
| *Calyptorhynchus banksii* |
| *Zanda funerea* |
| *Calyptorhynchus lathami* |
| *Caprimulgus macrurus* |
| *Carterornis leucotis* |
| *Casuarius casuarius* |
| *Centropus phasianinus* |
| *Cereopsis novaehollandiae* |
| *Certhionyx pectoralis* |
| *Certhionyx variegatus* |
| *Ceyx azureus* |
| *Peltohyas australis* |
| *Anarhynchus bicinctus* |
| *Charadrius dubius* |
| *Anarhynchus leschenaultii* |
| *Anarhynchus mongolus* |
| *Anarhynchus ruficapillus* |
| *Anarhynchus veredus* |
| *Chenonetta jubata* |
| *Cheramoeca leucosterna* |
| *Chlidonias hybrida* |
| *Chlidonias leucopterus* |
| *Chloris chloris* |
| *Chrysococcyx basalis* |
| *Chrysococcyx lucidus* |
| *Chrysococcyx minutillus* |
| *Chrysococcyx osculans* |
| *Pyrrholaemus sagittatus* |
| *Cincloramphus cruralis* |
| *Cincloramphus mathewsi* |
| *Cinclosoma castaneothorax* |
| *Cinclosoma castanotum* |
| *Cinclosoma cinnamomeum* |
| *Cinclosoma punctatum* |
| *Circus approximans* |
| *Circus assimilis* |
| *Cisticola exilis* |
| *Cisticola juncidis* |
| *Cladorhynchus leucocephalus* |
| *Climacteris affinis* |
| *Climacteris erythrops* |
| *Climacteris melanurus* |
| *Climacteris picumnus* |
| *Climacteris rufus* |
| *Colluricincla boweri* |
| *Colluricincla harmonica* |
| *Colluricincla megarhyncha* |
| *Colluricincla woodwardi* |
| *Columba leucomela* |
| *Conopophila albogularis* |
| *Conopophila rufogularis* |
| *Conopophila whitei* |
| *Coracina lineata* |
| *Coracina maxima* |
| *Coracina novaehollandiae* |
| *Coracina papuensis* |
| *Edolisoma tenuirostre* |
| *Corcorax melanorhamphos* |
| *Cormobates leucophaea* |
| *Corvus bennetti* |
| *Corvus coronoides* |
| *Corvus mellori* |
| *Corvus orru* |
| *Corvus tasmanicus* |
| *Coturnix pectoralis* |
| *Coturnix ypsilophora* |
| *Cracticus argenteus* |
| *Cracticus mentalis* |
| *Cracticus nigrogularis* |
| *Melloria quoyi* |
| *Gymnorhina tibicen* |
| *Cracticus torquatus* |
| *Cuculus optatus* |
| *Cyclopsitta diophthalma* |
| *Cygnus atratus* |
| *Dacelo leachii* |
| *Dacelo novaeguineae* |
| *Daphoenositta chrysoptera* |
| *Daption capense* |
| *Dasyornis brachypterus* |
| *Dasyornis broadbenti* |
| *Dendrocygna arcuata* |
| *Dendrocygna eytoni* |
| *Dendrocygna guttata* |
| *Dicaeum hirundinaceum* |
| *Dicrurus bracteatus* |
| *Diomedea exulans* |
| *Dromaius novaehollandiae* |
| *Drymodes brunneopygia* |
| *Drymodes superciliaris* |
| *Egretta garzetta* |
| *Egretta novaehollandiae* |
| *Egretta picata* |
| *Egretta sacra* |
| *Elanus axillaris* |
| *Elanus scriptus* |
| *Elseyornis melanops* |
| *Emblema pictum* |
| *Entomyzon cyanotis* |
| *Eolophus roseicapilla* |
| *Eopsaltria australis* |
| *Eopsaltria georgiana* |
| *Eopsaltria griseogularis* |
| *Ephippiorhynchus asiaticus* |
| *Epthianura albifrons* |
| *Epthianura aurifrons* |
| *Epthianura crocea* |
| *Epthianura tricolor* |
| *Poodytes carteri* |
| *Erythrogonys cinctus* |
| *Erythrotriorchis radiatus* |
| *Chloebia gouldiae* |
| *Esacus magnirostris* |
| *Eudynamys orientalis* |
| *Eudyptes pachyrhynchus* |
| *Eudyptula minor* |
| *Eulabeornis castaneoventris* |
| *Eurostopodus argus* |
| *Eurostopodus mystacalis* |
| *Eurystomus orientalis* |
| *Falco berigora* |
| *Falco cenchroides* |
| *Falco hypoleucos* |
| *Falco longipennis* |
| *Falco peregrinus* |
| *Falco subniger* |
| *Falcunculus frontatus* |
| *Fregata ariel* |
| *Fregata minor* |
| *Fulica atra* |
| *Fulmarus glacialoides* |
| *Gallinago hardwickii* |
| *Gallinago megala* |
| *Gallinula tenebrosa* |
| *Gavicalis fasciogularis* |
| *Gavicalis versicolor* |
| *Gavicalis virescens* |
| *Gelochelidon nilotica* |
| *Geoffroyus geoffroyi* |
| *Geopelia cuneata* |
| *Geopelia humeralis* |
| *Geophaps plumifera* |
| *Geophaps scripta* |
| *Geophaps smithii* |
| *Gerygone chloronota* |
| *Gerygone fusca* |
| *Gerygone levigaster* |
| *Gerygone magnirostris* |
| *Gerygone mouki* |
| *Gerygone olivacea* |
| *Gerygone palpebrosa* |
| *Gerygone tenebrosa* |
| *Glareola maldivarum* |
| *Gliciphila melanops* |
| *Glossopsitta concinna* |
| *Grallina cyanoleuca* |
| *Grantiella picta* |
| *Antigone antigone* |
| *Antigone rubicunda* |
| *Haematopus fuliginosus* |
| *Haematopus longirostris* |
| *Haliaeetus leucogaster* |
| *Haliastur indus* |
| *Haliastur sphenurus* |
| *Halobaena caerulea* |
| *Hamirostra melanosternon* |
| *Heteromunia pectoralis* |
| *Hieraaetus morphnoides* |
| *Himantopus himantopus* |
| *Hirundapus caudacutus* |
| *Hirundo neoxena* |
| *Hirundo rustica* |
| *Hydroprogne caspia* |
| *Hylacola cauta* |
| *Hylacola pyrrhopygia* |
| *Hypotaenidia philippensis* |
| *Irediparra gallinacea* |
| *Ixobrychus dubius* |
| *Ixobrychus flavicollis* |
| *Lalage leucomela* |
| *Larus dominicanus* |
| *Chroicocephalus novaehollandiae* |
| *Larus pacificus* |
| *Lathamus discolor* |
| *Leipoa ocellata* |
| *Leucosarcia melanoleuca* |
| *Lewinia pectoralis* |
| *Lichenostomus cratitius* |
| *Lichenostomus melanops* |
| *Lichmera indistincta* |
| *Calidris falcinellus* |
| *Limnodromus semipalmatus* |
| *Limosa lapponica* |
| *Limosa limosa* |
| *Lonchura castaneothorax* |
| *Lonchura flaviprymna* |
| *Lophochroa leadbeateri* |
| *Lophoictinia isura* |
| *Lopholaimus antarcticus* |
| *Machaerirhynchus flaviventer* |
| *Macronectes giganteus* |
| *Macronectes halli* |
| *Malacorhynchus membranaceus* |
| *Malurus amabilis* |
| *Malurus coronatus* |
| *Malurus cyaneus* |
| *Malurus elegans* |
| *Malurus lamberti* |
| *Malurus leucopterus* |
| *Malurus melanocephalus* |
| *Malurus pulcherrimus* |
| *Malurus splendens* |
| *Manorina flavigula* |
| *Manorina melanocephala* |
| *Manorina melanophrys* |
| *Manorina melanotis* |
| *Poodytes gramineus* |
| *Cincloramphus timoriensis* |
| *Megapodius reinwardt* |
| *Melanodryas cucullata* |
| *Meliphaga gracilis* |
| *Meliphaga lewinii* |
| *Meliphaga notata* |
| *Melithreptus albogularis* |
| *Melithreptus brevirostris* |
| *Melithreptus gularis* |
| *Melithreptus lunatus* |
| *Melopsittacus undulatus* |
| *Menura alberti* |
| *Menura novaehollandiae* |
| *Merops ornatus* |
| *Microcarbo melanoleucos* |
| *Microeca fascinans* |
| *Microeca flavigaster* |
| *Microeca griseoceps* |
| *Milvus migrans* |
| *Mirafra javanica* |
| *Monarcha frater* |
| *Monarcha melanopsis* |
| *Morus serrator* |
| *Motacilla flava* |
| *Myiagra alecto* |
| *Myiagra cyanoleuca* |
| *Myiagra inquieta* |
| *Myiagra rubecula* |
| *Myiagra ruficollis* |
| *Myzomela erythrocephala* |
| *Myzomela obscura* |
| *Myzomela sanguinolenta* |
| *Cinnyris jugularis* |
| *Aidemosyne modesta* |
| *Neochmia phaeton* |
| *Bathilda ruficauda* |
| *Neochmia temporalis* |
| *Neophema chrysogaster* |
| *Neophema chrysostoma* |
| *Neophema elegans* |
| *Neophema petrophila* |
| *Neophema pulchella* |
| *Neophema splendida* |
| *Neopsephotus bourkii* |
| *Nesoptilotis leucotis* |
| *Nettapus coromandelianus* |
| *Nettapus pulchellus* |
| *Ninox connivens* |
| *Ninox rufa* |
| *Ninox strenua* |
| *Northiella haematogaster* |
| *Numenius madagascariensis* |
| *Numenius minutus* |
| *Numenius phaeopus* |
| *Nycticorax caledonicus* |
| *Nymphicus hollandicus* |
| *Oceanites oceanicus* |
| *Ocyphaps lophotes* |
| *Onychoprion anaethetus* |
| *Oreoica gutturalis* |
| *Oreoscopus gutturalis* |
| *Origma solitaria* |
| *Oriolus flavocinctus* |
| *Oriolus sagittatus* |
| *Orthonyx spaldingii* |
| *Orthonyx temminckii* |
| *Oxyura australis* |
| *Pachycephala inornata* |
| *Pachycephala lanioides* |
| *Pachycephala melanura* |
| *Pachycephala olivacea* |
| *Pachycephala pectoralis* |
| *Pachycephala rufiventris* |
| *Pachycephala rufogularis* |
| *Pachycephala simplex* |
| *Pachyptila belcheri* |
| *Pachyptila desolata* |
| *Pachyptila salvini* |
| *Pachyptila turtur* |
| *Pandion haliaetus* |
| *Pardalotus punctatus* |
| *Pardalotus rubricatus* |
| *Pardalotus striatus* |
| *Parvipsitta porphyrocephala* |
| *Parvipsitta pusilla* |
| *Pedionomus torquatus* |
| *Pelagodroma marina* |
| *Pelecanoides urinatrix* |
| *Pelecanus conspicillatus* |
| *Peneothello pulverulenta* |
| *Petrochelidon ariel* |
| *Petrochelidon nigricans* |
| *Petroica boodang* |
| *Petroica goodenovii* |
| *Petroica phoenicea* |
| *Petroica rodinogaster* |
| *Petroica rosea* |
| *Petrophassa albipennis* |
| *Petrophassa rufipennis* |
| *Pezoporus wallicus* |
| *Phaethon lepturus* |
| *Phaethon rubricauda* |
| *Phalacrocorax carbo* |
| *Phalacrocorax fuscescens* |
| *Phalacrocorax sulcirostris* |
| *Phalacrocorax varius* |
| *Phaps chalcoptera* |
| *Phaps elegans* |
| *Phaps histrionica* |
| *Philemon argenticeps* |
| *Philemon buceroides* |
| *Philemon citreogularis* |
| *Philemon corniculatus* |
| *Calidris pugnax* |
| *Phoebetria palpebrata* |
| *Phonygammus keraudrenii* |
| *Phylidonyris niger* |
| *Phylidonyris novaehollandiae* |
| *Phylidonyris pyrrhopterus* |
| *Pitta iris* |
| *Pitta versicolor* |
| *Platalea flavipes* |
| *Platalea regia* |
| *Platycercus adscitus* |
| *Platycercus elegans* |
| *Platycercus eximius* |
| *Platycercus icterotis* |
| *Platycercus venustus* |
| *Plectorhyncha lanceolata* |
| *Plegadis falcinellus* |
| *Pluvialis fulva* |
| *Pluvialis squatarola* |
| *Podargus ocellatus* |
| *Podargus papuensis* |
| *Podargus strigoides* |
| *Podiceps cristatus* |
| *Poecilodryas cerviniventris* |
| *Poecilodryas superciliosa* |
| *Poephila acuticauda* |
| *Poephila cincta* |
| *Poephila personata* |
| *Poliocephalus poliocephalus* |
| *Polytelis alexandrae* |
| *Polytelis anthopeplus* |
| *Pomatostomus halli* |
| *Pomatostomus ruficeps* |
| *Pomatostomus superciliosus* |
| *Pomatostomus temporalis* |
| *Porphyrio porphyrio* |
| *Porzana fluminea* |
| *Zapornia pusilla* |
| *Zapornia tabuensis* |
| *Probosciger aterrimus* |
| *Procellaria aequinoctialis* |
| *Psephotellus chrysopterygius* |
| *Psephotellus dissimilis* |
| *Psephotus haematonotus* |
| *Psephotellus varius* |
| *Psitteuteles versicolor* |
| *Psophodes cristatus* |
| *Psophodes nigrogularis* |
| *Psophodes occidentalis* |
| *Psophodes olivaceus* |
| *Pterodroma cervicalis* |
| *Pterodroma lessonii* |
| *Pterodroma leucoptera* |
| *Pterodroma macroptera* |
| *Pterodroma mollis* |
| *Pterodroma nigripennis* |
| *Pterodroma solandri* |
| *Ptilinopus regina* |
| *Ptilinopus superbus* |
| *Chlamydera nuchalis* |
| *Ptilonorhynchus violaceus* |
| *Ptiloris magnificus* |
| *Ptilotula flavescens* |
| *Ptilotula fusca* |
| *Ptilotula keartlandi* |
| *Ptilotula ornata* |
| *Ptilotula penicillata* |
| *Ptilotula plumula* |
| *Puffinus assimilis* |
| *Puffinus gavia* |
| *Puffinus huttoni* |
| *Purnella albifrons* |
| *Purpureicephalus spurius* |
| *Pycnoptilus floccosus* |
| *Pyrrholaemus brunneus* |
| *Rallina tricolor* |
| *Ramsayornis fasciatus* |
| *Ramsayornis modestus* |
| *Recurvirostra novaehollandiae* |
| *Rhipidura albiscapa* |
| *Rhipidura dryas* |
| *Rhipidura leucophrys* |
| *Rhipidura phasiana* |
| *Rhipidura rufifrons* |
| *Rostratula benghalensis* |
| *Scenopoeetes dentirostris* |
| *Scythrops novaehollandiae* |
| *Sericornis beccarii* |
| *Neosericornis citreogularis* |
| *Sericornis frontalis* |
| *Sericornis keri* |
| *Sericornis magnirostra* |
| *Sericulus chrysocephalus* |
| *Smicrornis brevirostris* |
| *Sphecotheres vieilloti* |
| *Stagonopleura bella* |
| *Stagonopleura guttata* |
| *Stagonopleura oculata* |
| *Stercorarius antarcticus* |
| *Stercorarius parasiticus* |
| *Stercorarius pomarinus* |
| *Sterna dougallii* |
| *Sterna hirundo* |
| *Sterna paradisaea* |
| *Sterna striata* |
| *Sterna sumatrana* |
| *Sternula albifrons* |
| *Sternula nereis* |
| *Stictonetta naevosa* |
| *Stiltia isabella* |
| *Stipiturus malachurus* |
| *Stipiturus mallee* |
| *Stipiturus ruficeps* |
| *Stomiopera flava* |
| *Stomiopera unicolor* |
| *Strepera fuliginosa* |
| *Strepera graculina* |
| *Strepera versicolor* |
| *Struthidea cinerea* |
| *Acridotheres tristis* |
| *Sula dactylatra* |
| *Sula leucogaster* |
| *Syma torotoro* |
| *Symposiachrus trivirgatus* |
| *Tachybaptus novaehollandiae* |
| *Radjah radjah* |
| *Tadorna tadornoides* |
| *Tanysiptera sylvia* |
| *Thalassarche bulleri* |
| *Thalassarche carteri* |
| *Thalassarche cauta* |
| *Thalassarche chrysostoma* |
| *Thalassarche melanophris* |
| *Thalasseus bengalensis* |
| *Thalasseus bergii* |
| *Threskiornis molucca* |
| *Threskiornis spinicollis* |
| *Todiramphus chloris* |
| *Todiramphus macleayii* |
| *Todiramphus pyrrhopygius* |
| *Todiramphus sanctus* |
| *Tregellasia capito* |
| *Tregellasia leucops* |
| *Tribonyx ventralis* |
| *Trichodere cockerelli* |
| *Trichoglossus chlorolepidotus* |
| *Tringa brevipes* |
| *Tringa glareola* |
| *Tringa incana* |
| *Tringa nebularia* |
| *Tringa stagnatilis* |
| *Tringa totanus* |
| *Turnix melanogaster* |
| *Turnix pyrrhothorax* |
| *Turnix varius* |
| *Turnix velox* |
| *Tyto alba* |
| *Tyto longimembris* |
| *Tyto novaehollandiae* |
| *Tyto tenebricosa* |
| *Vanellus miles* |
| *Xanthotis flaviventer* |
| *Xanthotis macleayanus* |
| *Xenus cinereus* |
| *Zoothera heinei* |
| *Zoothera lunulata* |
| *Zosterops lateralis* |
| *Zosterops luteus* |
| *Acanthiza ewingii* |
| *Acanthornis magna* |
| *Melanodryas vittata* |
| *Melithreptus affinis* |
| *Melithreptus validirostris* |
| *Nesoptilotis flavicollis* |
| *Platycercus caledonicus* |
| *Sericornis humilis* |
| *Tribonyx mortierii* |
